# Potential applications of engineered bacteria in disease diagnosis and treatment

**DOI:** 10.20517/mrr.2024.57

**Published:** 2024-12-17

**Authors:** Zhaowei Luo, Zhanghua Qi, Jie Luo, Tingtao Chen

**Affiliations:** ^1^School of Huankui Academy, Nanchang University, Nanchang 330031, Jiangxi, China.; ^2^School of Public Health, Jiangxi Medical College, Nanchang University, Nanchang 330031, Jiangxi, China.; ^3^National Engineering Research Center for Bioengineering Drugs and the Technologies, Institute of Translational Medicine, Jiangxi Medical College, Nanchang University, Nanchang 330031, Jiangxi, China.; ^4^School of Pharmacy, Jiangxi Medical College, Nanchang University, Nanchang 330031, Jiangxi, China.; ^#^Authors contributed equally.

**Keywords:** Engineered bacteria, probiotics, synthetic biology, IBD, cancer, biosensors, mucosal vaccines

## Abstract

Probiotics are live microorganisms that confer health benefits to the host when administered in appropriate quantities. This beneficial effect has spurred extensive research in the medical and health fields. With rapid advancements in synthetic biology, the genetic and biological characteristics of a broad array of probiotics have been elucidated. Utilizing these insights, genetic editing technologies now enable the precise modification of probiotics, leading to the development of engineered bacteria. Emerging evidence underscores the significant potential of these engineered bacteria in disease management. This review explores the methodologies for creating engineered bacteria, their preliminary applications in healthcare, and the mechanisms underlying their functions. Engineered bacteria are being developed for roles such as *in vivo* drug delivery systems, biosensors, and mucosal vaccines, thereby contributing to the treatment, diagnosis, and prevention of conditions including inflammatory bowel disease (IBD), metabolic disorders, cancer, and neurodegenerative diseases. The review concludes by assessing the advantages and limitations of engineered bacteria in the context of disease management.

## INTRODUCTION

Probiotics are defined as “live microorganisms that, when administered in sufficient quantities, provide health benefits to the host”^[1]^. Research has increasingly focused on exploring the genetic structure and biological properties of diverse probiotics. Especially as the well-deserved hotspot, in-depth exploration of *Lactobacillus* and *Bifidobacterium* facilitates the identification and manipulation of crucial metabolic pathways and regulatory mechanisms^[2]^. Progressively clearer genetic backgrounds and inherent safety help make probiotics excellent vectors for engineered modifications.

Technological innovations in synthetic biology, high-throughput sequencing, omics, synthetic genomics, and other advanced fields have provided novel perspectives for the study of probiotics. Technologies like genetic programming and high-throughput sequencing enable the precise engineering of biological systems, analogous to techniques in electrical engineering^[3]^. Engineered bacteria are genetically modified to express exogenous genes or execute specific biological functions for treating, preventing, or diagnosing diseases^[4]^. Animal studies have demonstrated that engineered bacteria can influence a wide range of conditions including tumors^[5]^, inflammatory bowel disease (IBD)^[6]^, diabetes^[7]^, and neurodegenerative disorders^[8]^, spanning various categories such as infections, metabolism, and oncology. Some have progressed to clinical trials, such as *Escherichia coli* Nissle 1917 (EcN) engineered for colorectal cancer detection and treatment^[9]^. Engineered bacteria exhibit promising potential *in vivo*, leveraging both their inherent probiotic traits and engineered functionalities to enhance therapeutic efficacy.

This paper reviews current research on the applications and mechanisms of engineered bacteria for treating IBD, metabolic disorders, neurodegenerative conditions, and cancer. We also review the utilization of biosensors and mucosal vaccines built on engineered bacteria. Finally, we summarize the advantages and drawbacks of engineered bacteria in disease management.

## THE PREDECESSOR OF ENGINEERED BACTERIA - PROBIOTICS

Although many food supplements contain active microorganisms, they are categorized as probiotics only if they have been conclusively shown to benefit human health^[10]^. Common sources of widely used probiotics such as *Lactobacillus* and *Bifidobacterium* spp. include fermented foods, the human gut and vagina, and various plants^[2]^. Numerous animal studies have demonstrated that probiotics can enhance intestinal microecology, modulate host immunity and metabolism, provide resistance against infections, and potentially combat tumors. These effects depend on the probiotic itself or its secretions. For example, in the intestine, probiotics preempt the adhesion sites on intestinal epithelial cells through their adhesion proteins, thereby reducing the colonization of pathogenic bacteria^[11]^. Probiotics also secrete bacteriocins that inhibit the growth and proliferation of pathogenic bacteria such as *Listeria*, *Clostridium*, and *Salmonella* through mechanisms including pore formation, which disrupts the selective permeability of the target cell membrane^[12]^, interference with cell wall synthesis^[13]^, and enhancement of the host’s immune response^[14]^.

Notably, significant progress has been made in cancer therapy, such as the discovery that *Lactobacillus reuteri* ATCC BAA-2837 and its metabolite indole-3-aldehyde can enhance the effectiveness of immune checkpoint inhibitors in treating melanoma^[15]^. Recent clinical trials have also highlighted probiotics’ potential in managing conditions like anxiety and depression^[16]^, irritable bowel syndrome^[17]^, functional dyspepsia^[18]^, and postoperative constipation^[19]^. These findings underscore the promising role of probiotics in disease management. Strategies for microecological regulation of the human gut commonly include probiotic supplementation and fecal microbiota transplantation (FMT). However, these nonspecific approaches can have serious consequences due to the limited understanding of host-microbiota interactions. For instance, a patient with acute promyelocytic leukemia who consumed probiotic-enriched yogurt following diarrhea developed infectious shock one week later. The strain responsible for this adverse event was *Lactobacillus rhamnosus* GG^[20]^. This case is not unique; a study reported 48 clinical safety events related to probiotics between 2019 and 2021, including bacteremia, sepsis, and endocarditis^[21]^. These reports indicate that the safety of probiotics in immunocompromised hosts requires further evaluation. FMT, due to strict donor restrictions and inconsistent screening criteria across healthcare institutions, complicates the establishment of a unified risk profile, hindering its long-term clinical application^[22]^. Therefore, supplementation with probiotics and FMT may result in nonspecific adverse events, limiting their broader application. However, the development of engineered bacteria presents potential solutions to these challenges. While probiotics are natural candidates for engineering due to their beneficial properties, additional factors must also be considered. Researchers must also take into account factors such as colonization characteristics, ease of genetic modification, and available tools and techniques. In this context, *E. coli*, *Lactobacillus*, and *Bifidobacterium* spp. are considered promising chassis bacteria for genetic manipulation. For example, probiotic *Lactobacillus* species have been recognized as enerally recognized as safe (GRAS) after extensive use and investigation, providing a foundation for the subsequent development of food-grade carriers^[23]^. For instance, inducible plasmid self-destruction (IPSD)-assisted genome engineering can introduce homologous DNA into *Lactobacillus* and *Bifidobacterium* spp.^[24]^. The tetracycline-induced expression system and the CRISPR/Cas9 system facilitate precise control and editing of its genes^[25]^. These properties enable the continued advancement of probiotics as engineered microorganisms.

To avoid ambiguity, we hereby declare that the probiotics mentioned in this article refer only to strains that are known to be beneficial to the host, rather than including all strains indiscriminately. For example, when referring to “probiotics such as *Lactobacillus* and *Bifidobacterium* spp.” we are specifically referring to those species within these genera that have been proven to be beneficial to human health, and not to all species within the genera. Strains that are harmful or have not been confirmed to be beneficial are not within the scope of this discussion.

## POTENTIAL APPLICATIONS OF ENGINEERED BACTERIA IN DISEASE MANAGEMENT

The rapid advancement of synthetic biology technologies^[26]^ has facilitated their transition from laboratory settings to clinical trials, driving research toward *in vivo* therapeutics using engineered bacteria in the 21st century. Our review of engineered bacteria underscores their crucial role in disease management involving treatment, diagnosis, and prevention.

### Potential applications of *in vivo* drug delivery systems developed based on engineered bacteria in disease treatment

**Figure 1:**
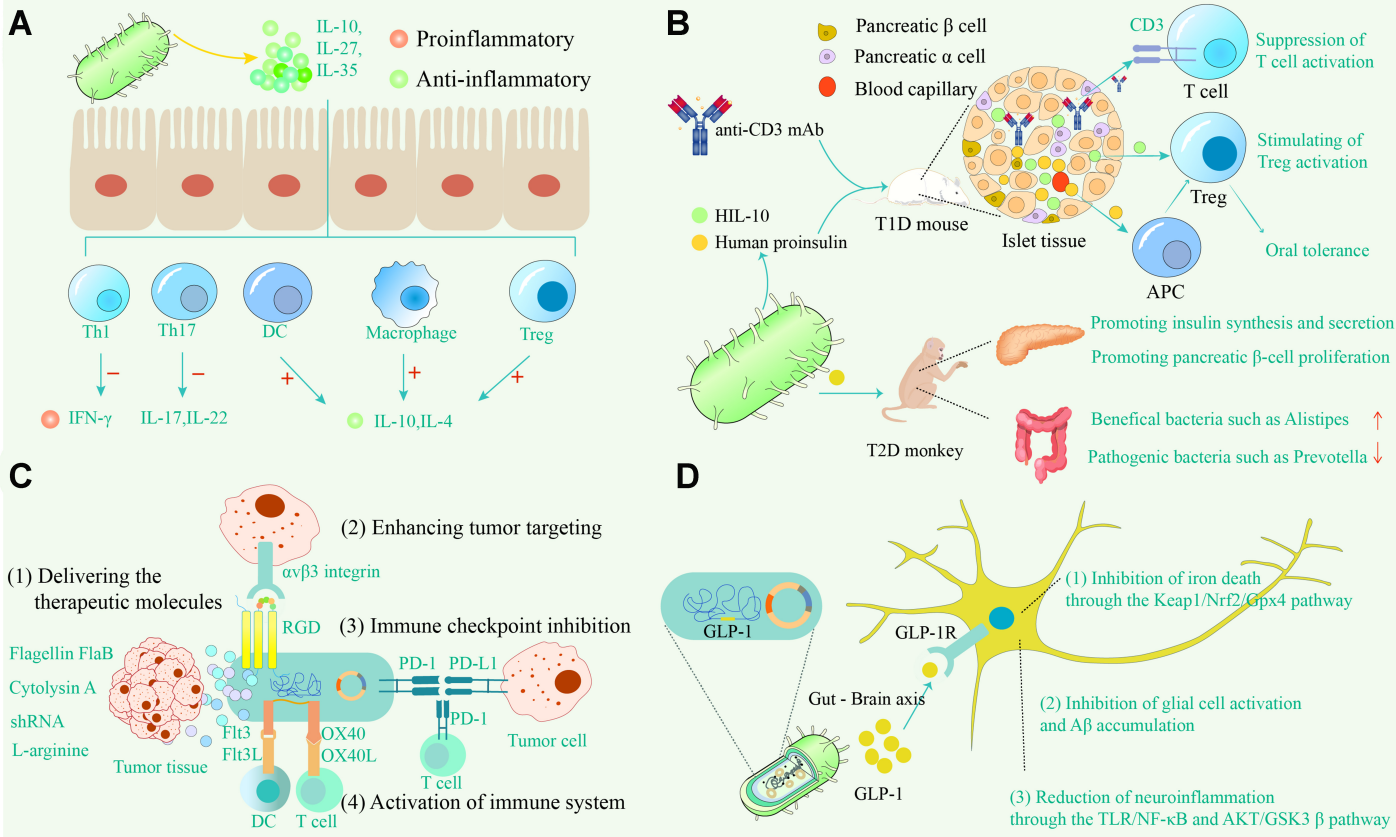
Mechanistic insights into the application of engineered bacterial-based *in vivo* drug delivery systems for disease therapy. (A) Intestinal *in situ* production of anti-inflammatory mediators; (B) Diabetes therapy in animal models; (C) Antitumor strategy; (D) Neurodegenerative disease intervention.

As traditional drug delivery systems struggle to match the pace of disease progression, emerging methods are increasingly favored. The short half-life, high toxicity, and expense of certain drugs necessitate novel and more efficient delivery systems. Genetic engineering of probiotics enables precise modulation of heterologous protein expression, facilitating efficient production of target proteins, peptides, and small-molecule metabolites, among others. *In vivo* drug delivery systems utilizing these engineered bacteria have demonstrated significant potential in treating IBD, metabolic disorders, neurodegenerative disease, cancer, and various other diseases [Figure 1].

#### IBD

IBD encompasses chronic, nonspecific inflammatory conditions affecting the intestines, notably Crohn’s disease (CD) and ulcerative colitis (UC)^[27]^. IBD is a global health issue with a complex pathogenesis that involves genetic, immunologic, and microbial factors^[28]^. From a microbiological standpoint, the disrupted intestinal mucosal barrier in UC, caused by an imbalanced intestinal microecology, leads to inappropriate immune system activation and subsequent tissue damage^[29]^. Additionally, reactive oxygen species (ROS) and protein hydrolases released by neutrophils during intestinal inflammation can disrupt tight junctions between intestinal epithelial cells, further compromising the intestinal barrier^[30]^. Furthermore, short-chain fatty acids, which are metabolites of intestinal microorganisms, have been shown to regulate the quantity and function of Tregs, thereby modulating the immune status of the gut^[31]^.

Improving the inflammatory state of the intestine is a key focus in the treatment of IBD. Traditional therapies typically involve the use of anti-inflammatory drugs and immunosuppressive agents, such as anti-TNF-α antibodies. Excessive exposure of active drugs to the intestinal lumen can lead to their absorption into the systemic circulation through the intestinal mucosa, potentially triggering adverse reactions^[32]^. To minimize unnecessary drug exposure and related side effects, an *in vivo* drug delivery system based on engineered bacteria has been developed, which enables targeted colonization^[33]^. In 2000, Steidler *et al.* first reported the use of engineered *Lactococcus lactis* to deliver mIL-10 for the treatment of IBD in mice^[34]^. In this study, the *mIL-10* gene sequence was cloned into a plasmid and introduced into *Lactococcus lactis* via electrotransformation, enabling the bacteria to produce mIL-10. However, the potential accumulation of transgenic strains raised biosafety concerns. To address this, Steidler *et al.* utilized a clever biocontainment strategy: they constructed a homology arm containing the upstream and downstream regions of the *thyA* gene, which, when introduced into *Lactococcus lactis* along with an hIL-10 expression cassette, allowed for the replacement of *thyA* with hIL-10 through double homologous recombination^[35]^. Subsequent clinical trials confirmed the efficacy of both the biocontainment strategy and the therapeutic approach^[36]^.

**Figure 2:**
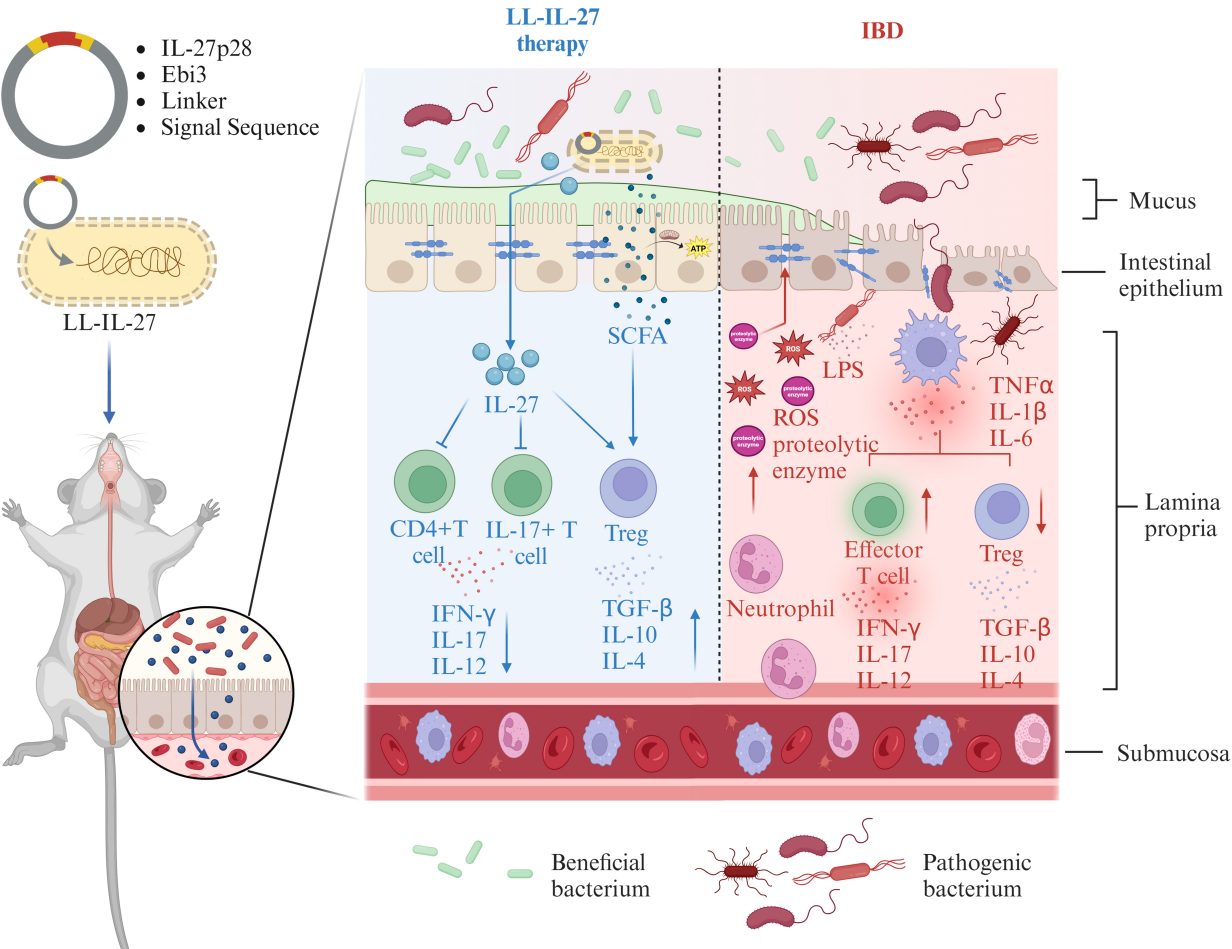
Mechanism of intestinal delivery of IL-27 by engineered LL-IL-27 for the treatment of IBD (Created with https://BioRender.com). IBD: Inflammatory bowel disease.

To ensure efficient delivery of target molecules to lesions, engineered bacteria often require modifications to their expression plasmids, such as codon optimization and the addition of secretion signal sequences. Codon optimization involves altering codons in exogenous gene sequences to align with the host cell's preferred codon usage, thereby enhancing translation efficiency. This strategy has been widely applied in the design of engineered bacteria^[37]^. The process relies heavily on bioinformatics in synthetic biology, alongside advances in molecular biology^[38]^. Another approach to improving the delivery of heterologous proteins by engineered bacteria is the incorporation of a secretion signal sequence at the N-terminal of the target gene. For instance, in a study by Hanson *et al.*, the genes encoding two key subunits of IL-27, IL-27p28 and Ebi3, were codon-optimized and fused by a contiguous sequence, with a secretion signal sequence added at the N-terminus. The engineered *Lactococcus lactis* (LL-IL-27) exhibited enhanced IL-27 delivery capacity. This modification made LL-IL-27 more effective in treating IBD in mice compared to both LL-IL-10 and systemically administered recombinant IL-27 [Figure 2]. LL-IL-27 showed efficacy by stimulating IL-10 production in T cells, reducing CD4^+^ T cells among intestinal lymphocytes, and mitigating inflammatory cell infiltration^[39]^.

The gastrointestinal environment in IBD presents a complex challenge, as even engineered bacteria that are optimized for safety and production efficiency may be compromised before they reach their target site for colonization. To enhance the bioavailability of these bacteria, researchers have combined them with various biomaterials to create effective drug delivery systems. For example, in a study focused on removing ROS from the IBD gut, researchers used two biocompatible and biodegradable materials - chitosan and sodium alginate - to coat engineered EcN expressing catalase (CAT) and superoxide dismutase (SOD) via a layer-by-layer electrostatic self-assembly technique, resulting in EcN-pE(C/A)2. These biomaterials protected the engineered bacteria from digestive enzymes and the acidic conditions of the gastrointestinal tract, while also enhancing their compatibility with other materials. This strategy improved the stability and bioavailability of the engineered bacteria^[40]^. Interestingly, EcN can also be designed as an inactive carrier for drug delivery. Researchers introduced the lysin E gene from phage φX174 into EcN, inducing its expression to cause the bacterium to lyse into an empty shell that retains only the surface structure. These “EcN ghosts” have demonstrated safety and efficacy in IBD treatment^[41]^.

To conclude, engineered bacteria function as carriers for localized delivery of live immunoreactive substances, effectively bypassing systemic symptoms and addressing the short half-life concerns of direct cytokine delivery. Future advancements in modulating probiotic genes show significant promise for optimizing this *in vivo* drug delivery system for treating IBD.

#### Metabolic disease

Diabetes Type 2 diabetes (T2D) is characterized by insulin resistance initially, leading to impaired glucose utilization by tissue cells and eventual pancreatic β-cell dysfunction. Dysregulation of the host’s gut microbiota has been implicated in T2D development, impacting metabolism, inflammatory responses, and oxidative stress^[42,43]^. T2D patients often exhibit an imbalance with increased pathogenic bacteria and reduced butyric acid-producing bacteria in the gut, influencing host immune and metabolic functions^[42,44]^. Further clinical and animal studies have demonstrated that probiotic supplementation with strains such as *Lactobacillus acidophilus*, *Bifidobacterium lactis*, and *Lactobacillus casei* Shirota can modestly lower blood glucose levels in T2D patients^[45,46]^, suggesting a potential role for probiotics in T2D management.

On another front, glucagon-like peptide-1 (GLP-1), a newly discovered gastrointestinal hormone, stimulates insulin secretion and inhibits glucagon secretion, thereby lowering blood glucose levels^[47,48]^. However, its short *in vivo* half-life limits clinical efficacy^[49]^. *In vivo* drug delivery systems based on engineered bacteria offer a promising solution to this challenge. As mentioned earlier, probiotics have demonstrated significant potential in the treatment of T2D, making them ideal candidates as chassis bacteria for the development of engineered strains. Engineered strains such as *Lactococcus lactis* (LL-pUBGLP-1)^[50]^ and *Lactobacillus plantarum* (*L. plantarum*-pMG36e-GLP-1)^[7,51]^, transformed with plasmids containing GLP-1 cDNA, can effectively produce and deliver GLP-1 orally, significantly enhancing its therapeutic potential. In animal models and clinical trials, oral administration of *Lactobacillus plantarum*-pMG36e-GLP-1 reduced pathogenic bacteria such as *Prevotella* and increased beneficial butyrate-producing *Alistipes* spp. in the gut of spontaneous T2D monkeys, improving intestinal dysbiosis^[51]^ [Figure 1B]. Additionally, *Lactobacillus plantarum*-pMG36e-GLP-1 has shown efficacy in mitigating high-fat diet-induced obesity in mice by promoting fatty acid oxidation and modulating intestinal flora^[52]^. In addition, engineered *Lactococcus lactis* strains for the treatment of type 1 diabetes (T1D) are under development. For example, an engineered strain of *Lactococcus lactis* that secretes the intact insulin autoantigen and the immunomodulatory cytokine IL-10 has been shown to successfully cure diabetes in non-obese diabetic (NOD) mice when used in conjunction with low-dose systemic anti-CD3 antibody therapy^[53]^.

*E. coli* Nissle 1917, a probiotic, exerts influence on glycemic responses through intricate host interactions beyond mere glucose uptake^[54]^. Like probiotic *Lactobacilli*, *E. coli* Nissle 1917 can be genetically engineered to produce a GLP-1-like product locally, exerting beneficial effects in managing diabetes. Researchers have proposed expressing a cholera toxin B subunit-insulin-like growth factor 1 fusion protein (CTB-IGF-1) using engineered *E. coli* Nissle 1917. Here, the cholera toxin B subunit (CTB) serves as a carrier protein facilitating the targeting of insulin-like growth factor 1 (IGF-1), which mimics insulin's physiological function via distinct receptors^[55]^. While this hypothesis has not been confirmed by subsequent reports, analogous studies have been conducted. For instance, engineered *E. coli* BL21 expressing a CTB-10×rolGLP-1 fusion protein significantly lowered blood glucose levels in mice with T2D^[56]^. Moreover, engineered *E. coli* BL21 (DE3) expressing β-cell expansion factor A (BefA) holds promise for T2D treatment by enhancing pancreatic β-cell proliferation while mitigating inflammation and apoptosis^[57,58]^.

Phenylketonuria Phenylketonuria (PKU) is an inherited metabolic disorder caused by autosomal recessive inheritance, mainly resulting from a deficiency of phenylalanine hydroxylase (PAH) in patients. Additionally, the PAH-catalyzed conversion of phenylalanine (Phe) to tyrosine (Tyr) requires tetrahydrobiopterin (BH4) as a cofactor. Therefore, patients with impaired BH4 metabolism may also develop PKU^[59]^. This deficiency leads to the excessive accumulation of phenylalanine, which can damage the nervous system^[60]^. Historically, a strict low-Phe diet has been the cornerstone of PKU treatment^[61]^. However, long-term dietary control does not alter the underlying nature of the disease. This approach is not only difficult to implement but may also lead to other complications^[62]^. Recently, new treatment protocols such as gene therapy^[63]^ and enzyme replacement therapy^[64]^ have been proposed and validated. Engineered bacteria could potentially enhance enzyme replacement therapy for PKU.

Enzymes currently under study for treating PKU include phenylalanine lyase (PAL) and PAH, both crucial for reducing Phe levels via metabolic pathways^[64,65]^. The Anabaena variabilis phenylalanine ammonia lyase gene (*AvPAL*) was optimized and cloned into *Lactobacillus reuteri* 100-23C. The engineered bacterium demonstrated a significant reduction in blood Phe levels in mice within 3 to 4 days of administration^[66]^. AvPAL functions by breaking down accumulated Phe into cis-cinnamic acid and ammonia, which is excreted through normal metabolic pathways. In contrast, PAH can be secreted into the intestines by engineered *Lactobacillus plantarum* CM_PUJ411, detected using the fluorescent protein. This strain operates through two primary mechanisms: secreted PAH catalyzes the conversion of L-phenylalanine (L-Phe) to L-tyrosine (L-Tyr), which is then excreted via normal metabolic pathways. Additionally, it expresses a cell-penetrating peptide, the TAT protein transduction domain, which enhances the intestinal uptake efficiency of recombinant PAH and potentially reduces degradation within the intestinal lumen^[67]^. The construction of the two types of engineered bacteria mentioned above involves two common plasmid-based expression systems: the constitutive expression system and the inducible expression system. Typically, plasmid-based expression systems include key elements such as a replicon, a promoter with a ribosome binding site and regulatory sequences, a terminator, a target gene, and a selection marker. In a previous project, a plasmid-based expression system was constructed in *Anabaena variabilis*, where the promoter of the *AvPAL* gene was replaced with a high-yield constitutive promoter from *Lactobacillus casei* (the erythromycin resistance B gene, *ermB*), enabling sustained and stable expression of PAL in the host cells. PAL expression in the host cell does not require additional inducible signals. In contrast, the pSIP503 expression vector carrying the *PAH* gene features a nisin-inducible promoter, which drives the transcription of downstream target genes when nisin levels in the environment are elevated. Both constitutive expression systems (e.g., pUBU^[68]^, pPBT-GFP^[69]^, pNZ2103^[70]^) and inducible systems (e.g., pMSP3535^[71]^, pULP3-PLDH^[72]^, pMY01^[73]^) offer distinct advantages. Constitutive systems enable stable and efficient expression of therapeutic proteins in complex intestinal environments, while inducible systems are better suited for industrial-scale recombinant protein production. However, a significant drawback of plasmid-based expression systems is the biosafety concern related to the antibiotic resistance genes they often contain. Both the U.S. Food and Drug Administration (FDA) and the European Food Safety Authority (EFSA) have stated that food-grade vectors must be free of antimicrobial resistance elements. Fortunately, alternative methods exist to eliminate antibiotic resistance genes, such as gene editing using the λ Red recombination system. This system typically involves three key proteins: the Exo protein, which degrades single-stranded DNA; the β protein, which facilitates homologous recombination; and the γ protein, which helps convert linear DNA into a circular form for incorporation into the chromosome. Isabella *et al.* selected the genes for PAL and L-amino acid deaminase (LAAD) as the target genes for Phe degradation and integrated them into the EcN chromosome using the λ Red system. A special plasmid was then introduced to recognize and excise the expression cassette containing the antibiotic resistance genes, using flippase (FLP) recombinase. The expression of the *PAL* and *LAAD* genes was controlled by the hypoxia-inducible promoter PfnrS, allowing for gene expression in the hypoxic conditions of the intestine. The engineered bacterium, SYNB1618, was shown to reduce Phe levels in mice and rhesus monkeys by converting Phe into trans-cinnamic acid, which is subsequently metabolized into uric acid and excreted by the host^[74]^.

Several engineered bacterial strains, including TYS8500^[75]^, SYNB1618^[74,76]^, and SYNB1934^[77,78]^, utilize EcN as a vector to express PAL effectively, thereby improving Phe degradation rates. SYNB1618 demonstrated safety and tolerability in clinical trials, achieving a maximum tolerated dose of 2 × 10^11^ colony-forming units^[76]^. SYNB1934, optimized for PAL enzyme activity through directed evolution, exhibited enhanced enzyme efficacy and stability, alongside favorable safety and pharmacokinetic profiles in non-human primate models^[78]^. These studies propose innovative approaches for PKU treatment using engineered bacteria as a therapeutic strategy.

#### Cancer

Historically, tumor regression has been observed in association with local infections^[79]^. Advances in understanding the human microbiome, particularly the concept of intratumoral microbiota, highlight the critical role of bacteria in tumor growth. Previous studies have shown that certain bacteria can target and colonize tumor tissue, playing a role in the formation of the tumor microenvironment. For example, EcN, attenuated *Salmonella typhimurium*, and *Lactobacillus paracasei* have demonstrated this ability^[80-82]^. Tumor colonization can occur passively, such as when vascular disruption, caused by a sudden increase in TNF-α in the tumor vasculature, leads to bacterial influx^[83]^. Additionally, *Salmonella typhimurium* strains lacking certain chemotactic receptors (e.g., tar, tsr, trg receptors) lose their ability to colonize tumors, suggesting that specific chemical signals in tumor tissues may guide bacterial colonization^[84]^. Furthermore, the low-oxygen environment of tumor tissues may attract anaerobic bacteria, supporting their colonization. Bacteria colonizing tumor tissues can enhance the body’s ability to fight tumors by reprogramming the tumor microenvironment, particularly through the modulation of immune cells^[85]^. For example, *Lactococcus lactis* subsp. *cremoris* C60 induces a macrophage inflammatory phenotype via TLR signaling, which promotes antigen-dependent activation of tumor-specific CD8^+^ T cells, thereby enhancing the immune response against melanoma^[86]^. Additionally, *Lactobacillus plantarum* L168 and its metabolite indole-3-lactic acid (ILA) stimulate IL-12 production by dendritic cells (DCs), leading to CD8^+^ T cell activation and improved outcomes in colorectal cancer.

In summary, the specific mechanisms through which natural bacteria target and colonize tumors remain under investigation. However, these bacteria are already promising candidates for chassis engineering due to their potential therapeutic advantages. Before such applications can be realized, the safety concerns surrounding certain bacteria, such as *Salmonella typhimurium*, need to be addressed. *Salmonella typhimurium* is a Gram-negative, foodborne pathogen that causes gastrointestinal symptoms and systemic sepsis^[87]^. In 1999, Low *et al.* made the first attempt to modify the virulence of *Salmonella typhimurium* by removing the *msbB* gene, which is involved in lipopolysaccharide synthesis^[88]^. They introduced the knockout vector pDBMS7, which contains an incomplete fragment of the *msbB* gene, into *Salmonella typhimurium* strain YS501 and used bacterial homologous recombination to replace the wild-type *msbB* gene. This resulted in attenuated virulence, which was confirmed in mice and pigs. In a similar approach, Clairmont *et al.* deleted the *purI* gene, an essential enzyme in the purine synthesis pathway, from the *Salmonella* genome, creating a purine-deficient strain, VNP20009. This modification limited the bacteria’s undesirable bioaccumulation and enhanced its safety profile^[89]^. The VNP20009 strain demonstrated some safety in subsequent Phase I clinical studies, although dose-related toxicity was observed, and no significant antitumor effects were noted^[81]^. Another strategy involves targeting *Salmonella pathogenicity island 1* (*SPI1*), a key virulence factor of *Salmonella*, whose expression is regulated by ppGpp. Specifically, ppGpp modulates *SPI1* gene expression by activating hilA through the activity-dependent pathway of SpoT^[90]^. Knocking out the relA and *spoT* genes in *Salmonella typhimurium* resulted in the ΔppGpp strain, which proved to be nearly non-toxic in mice, showing potential as a vaccine vector^[91]^.

Once the safety concerns are addressed, it will be essential to improve the tumor-targeting and colonization capabilities of engineered bacteria. Although candidate chassis bacteria have demonstrated significant colonization abilities in their natural states, the underlying mechanisms are still largely unknown and challenging to predict. One promising approach to enhance tumor targeting is the incorporation of tumor-targeting molecules onto the surface of engineered bacteria through synthetic biology. This could significantly increase their ability to home in on tumor tissues^[92]^. For example, the RGD peptide sequence, a known ligand for αvβ3 integrin, which is overexpressed on various tumor cells and endothelial cells during tumor angiogenesis, has been used to enhance targeting. By inserting the RGD peptide sequence into the outer membrane protein A of the attenuated *Salmonella* strain ΔppGpp, the resulting ΔppGppRGD strain exhibited strong binding to αvβ3-overexpressing cancer cells and demonstrated efficient targeting of αvβ3-expressing tumor xenografts *in vivo*^[93]^. Similarly, an engineered strain called 2G9-*Salmonella* was designed to specifically infect CD20-positive tumor cells. This was achieved by displaying a single-domain antibody (VHH) targeting the CD20 antigen on the outer membrane of the SL3261 aroA-deficient *Salmonella* strain^[94]^.

Drug molecules or protein molecules with antitumor effects carried by engineered bacteria can effectively kill tumors. For example, the surface of EcN was coupled with the anticancer drug doxorubicin through pH-sensitive amide bonds, and then ligand-linked with photosensitizer gold nanorods (AuNRs) to form EcN-Dox-Au microrobots. With the assistance of NIR laser, the AuNRs produced a photothermal effect, which enhanced the permeability of the tumor cell membrane and promoted the microrobots to deep tumor. The micro-robots penetrated into the deep tumor tissues. Then, doxorubicin was released under the acidic condition of the tumor microenvironment to kill the tumor^[95]^. In another type of scenario, the engineered bacteria are designed to express proteins with antitumor effects that act in various ways, including activating the host immune system, inducing apoptosis, and interfering with tumor cell metabolism. For example, the Flt3L and OX40L fusion protein expressed by FOLactis promotes DC maturation and T cell activation, converting cold tumors into hot tumors^[96]^. In addition, attenuated *Salmonella typhimurium* VNP20009 activates T cells by displaying mPD-1 at the membrane and relieves tumor cells from immune escape^[97]^. *Lactococcus lactis* has been genetically modified to carry the gene encoding tumor necrosis factor-related apoptosis-inducing ligand (TRAIL), overcoming its biological half-life limitations and demonstrating apoptosis induction in colon cancer cells *in vitro*^[98]^. In addition, engineered *Salmonella typhimurium* exhibited inhibitory effects on a variety of cancers, including melanoma, colon and pancreatic cancers, by delivering molecules such as shRNA targeting the inhibin α-subunit (INHA)^[99]^, heterotrimeric flagellin FlaB^[100]^, and cytolysin A (ClyA)^[101]^ to the tumor tissues.

In a colon cancer mouse model, a combination therapy involving L-arginine and anti-PD-1 antibodies significantly improved survival rates^[102]^. To enhance the precise and efficient delivery of L-arginine to tumor tissues, an engineered strain of EcN was developed, demonstrating high efficiency in L-arginine production and synergistically enhancing the effects of anti-PD-1 antibody treatment^[103]^.

#### Neurodegenerative disease

Neurodegenerative diseases such as Alzheimer’s disease (AD) and Parkinson’s disease (PD) are characterized by progressive neuronal loss in the brain and spinal cord, leading to chronic neurological dysfunction and cognitive or motor decline. Their pathogenesis encompasses intricate factors including neuroinflammation, insulin resistance, mitochondrial dysfunction, and protein misfolding and aggregation^[104]^. GLP-1, originally recognized for its therapeutic role in metabolic disorders such as diabetes and obesity, has now gained prominence in the realm of neurodegenerative diseases. Studies have demonstrated its ability to mitigate neuroinflammation^[105]^, improve insulin sensitivity^[106]^, confer neuroprotective effects^[107]^, and preserve mitochondrial function^[108]^. Engineered bacteria represent a promising strategy to tackle the obstacles of GLP-1 therapy. By synthesizing GLP-1 *in situ* within the gut, they can circumvent its limited half-life. Moreover, probiotics exert influence on the central nervous system by modulating metabolism and immunity *via* the gut-brain axis^[109]^. Therefore, the combination of probiotics and GLP-1 is anticipated to play an increasingly significant role in the treatment of neurodegenerative diseases.

In a previous study, we constructed the engineered bacterial plasmid pMG36e-GLP-1 containing the *GLP-1* gene, which was subsequently transformed into *Lactococcus lactis* to generate the engineered strain MG1363-pMG36e-GLP-1. Administering MG1363-pMG36e-GLP-1 orally to mice afflicted with LPS-induced systemic inflammation led to decreased inflammation, substantial enhancements in spatial learning and memory, and suppression of glial cell activation and Aβ accumulation^[110]^. Subsequent investigations utilized MG1363-pMG36e-GLP-1 in a murine model of MPTP-induced PD, revealing significant enhancements in motor function, reduced dopaminergic neuron loss, diminished α-synuclein aggregation, and suppression of ferroptosis through activation of the Keap1/Nrf2/GPX4 pathway^[111,112]^. Moreover, MG1363-pMG36e-GLP-1 modulates the TLR4/NF-κB and AKT/GSK3β pathways to reduce the expression of inflammatory factors and boost neuroprotective factor activity, thereby notably improving spatial learning and memory in AD mice^[8]^.

### Potential applications of biosensors developed based on engineered bacteria for disease diagnosis

**Figure 3:**
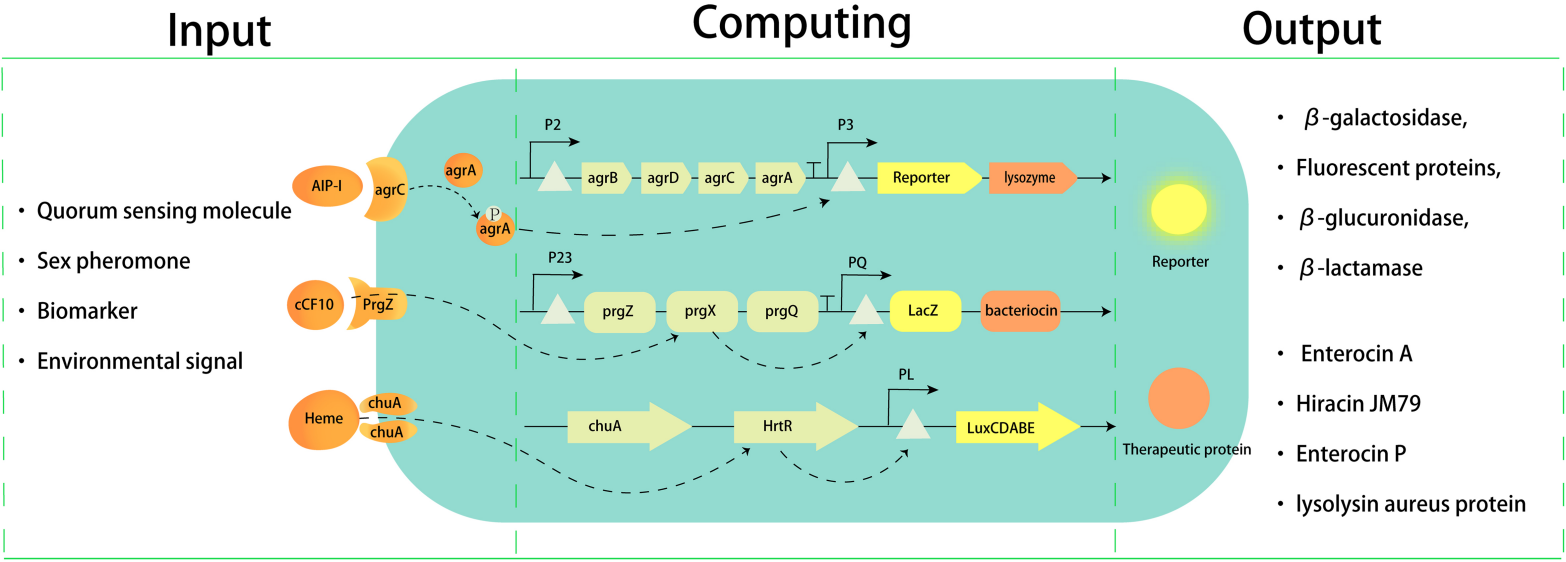
Schematic of the working principle of a biosensor constructed using engineered bacteria.

Engineered bacteria designed as biosensors represent an innovative approach in biotechnology, particularly for detecting pathogenic bacteria via their quorum sensing (QS) systems. QS involves bacterial communication via chemical signaling molecules, facilitating coordinated behavioral changes based on population density and surrounding species^[113]^. These biosensors utilize QS molecules as input modules, computational gene networks for processing, and output modules, such as reporter genes (e.g., β-galactosidase, fluorescent proteins, β-glucuronidase, β-lactamase), for detection [Figure 3].

For instance, researchers integrated the agrQS system from *Staphylococcus aureus* into *Lactobacillus reuteri* DSM20016 to detect autoinducing peptide I (AIP-I), a QS molecule produced by *Staphylococcus aureus*^[114]^. This engineered bacterium can sense AIP-I concentration-dependently by employing reporter genes such as *GusA*, which is linked to the production of a yellow pigment. Upon AIP-I binding to AgrC, it initiates AgrA phosphorylation, resulting in GusA expression repression and thereby indicating AIP-I presence indirectly through pigment production^[115]^. Li *et al.* expanded on this concept by engineering *Lactobacillus plantarum* WCSF I with an agrQS system for detecting AIP-I. Initially employing GFP as a reporter for fluorescence-based detection, they subsequently modified the system to secrete lysozyme upon AIP-I detection, creating a dual-function biosensor that detects and inhibits *S. aureus* growth^[116]^.

Additionally, Mao *et al.* engineered *Lactococcus lactis* to detect Vibrio cholerae using a hybrid receptor HR capable of sensing V. cholerae’s population sensing molecule CAI-1. They utilized fluorescent and colorimetric reporter genes such as mCherry and β-lactamase for *in vitro* and *in vivo* detection, showcasing the versatility of engineered bacteria in diverse detection environments^[117]^. While the sex pheromone of certain Gram-negative bacteria can serve as a detection signal, Borrero *et al.* utilized the *Enterococci* sex pheromone, cCF10, to construct a pCF10-based expression vector introduced into *Lactococcus lactis* NZ9000 for detecting *Enterococci* presence^[118]^. The development of these biosensors illustrates the significant potential of engineered *Lactococcus lactis* in pathogenic bacteria monitoring.

It is evident that mere detection of pathogenic bacteria is insufficient; there is a desire to extend biosensor capabilities to detect biomarkers that hold greater significance in disease diagnosis. Biomarkers encompass various characteristics (such as cells, proteins, molecules, and genes) measurable from biological samples. Their presence or fluctuation can indicate the status or progression of specific diseases, playing pivotal roles in disease prevention, diagnosis, treatment, detection, and prognosis assessment^[119]^. This expectation has been partially realized in biosensors utilizing probiotics as chassis, exemplified by Mimee *et al.* They integrated hemoglobin-sensing, genetically modified EcN with ultra-low-power microelectronics to develop an ingestible micro-bio-electronic system (IMBED). This system detects luminescence signals from bacteria and wirelessly transmits them to external devices, effectively detecting gastrointestinal hemorrhage in mouse and pig models^[120]^. In the context of gut diseases like IBD, genetically engineered EcN, known as i-ROBOT, has been pivotal. Leveraging CRISPR-Cas9 gene editing technology, i-ROBOT detects inflammatory marker thiosulfate levels, expressing associated fluorescent proteins to diagnose IBD sensitively^[121]^. Similarly, calreticulin serves as another non-invasive IBD marker^[122]^. For tumor diagnostics, engineered EcN expressing tumor biomarker nitroreductase (NTR) has shown promise. Exploiting its natural tumor tropism, EcN stably expresses NTR in tumor tissues, activating fluorescent probes for tumor imaging, thereby aiding in tumor diagnosis and surgical resection^[123]^. Similarly, EcN was engineered to express β-galactosidase in tumor cells, which subsequently degrades the injected substrate LuGal, resulting in the production of fluorescein detectable in the urine^[124]^. These studies have demonstrated non-invasive, non-radioactive approaches for the early detection of tumors. These engineered bacteria are referred to as bacterial whole-cell biosensors (BWCBs)^[125]^. In the context of disease detection, BWCBs offer advantages such as reduced reliance on expensive equipment, faster detection, greater specificity, and lower costs, which facilitate the early diagnosis of certain diseases. Furthermore, engineered bacteria can be designed as imaging vectors to improve the sensitivity and specificity of medical imaging. Additionally, integrated diagnostic and therapeutic engineered bacteria are being developed to concurrently release therapeutic drugs while diagnosing disease markers^[116]^.

### Potential application of mucosal vaccines developed based on engineered bacteria in disease prevention

Mucosal surfaces in the respiratory, digestive, and genitourinary systems serve as crucial entry points for pathogens from the environment. The mucosal immune system plays a pivotal role as the body’s initial defense barrier against these pathogens. Therefore, effective vaccines that stimulate immune responses at mucosal sites are vital.

Research dating back to the early 1990s has underscored the potential of *Lactobacillus casei* in enhancing mucosal immune responses. For example, studies have shown that *Lactobacillus casei* can protect against *Salmonella typhimurium* infection by boosting the production of sIgA in intestinal mucosal secretions^[126]^. This highlights the ability of natural *Lactobacillus casei* to serve as an immune adjuvant for preventing intestinal infections.

Recent advancements in probiotic genomics and synthetic biology have significantly advanced the design of engineered bacteria. These probiotics are engineered to express antigenic components from various pathogens such as bacteria^[127]^, viruses^[128]^, and parasites^[129]^. Administering these engineered bacteria mucosally can stimulate immune responses within the mucosal immune system, thereby exerting anti-infective effects targeted against specific pathogens.

Several recent animal studies have demonstrated the superior immunoprotective effects of engineered bacteria against pathogens such as *Streptococcus pneumoniae*^[127]^, *Trichinella spiralis*^[129]^, *Helicobacter pylori*^[130,131]^, *Avian influenza A* virus^[132]^, and COVID-19^[133]^. Various factors influence the immunization efficacy of engineered bacteria, including chassis type, antigen expression site, administration route, and target antigen type. A study examined the impact of varying antigen anchoring positions on immunization efficacy. They introduced two plasmids, pPG1 (surface display) and pPG2 (secretion), into the probiotic strain *Lactobacillus casei* CC16 for oral administration to carp, aiming to stimulate mucosal immune responses against *Aeromonas hydrophilia.* They discovered that surface-displayed Lc-pPG1-Aha1 induced higher levels of specific antibodies and enhanced leukocyte phagocytosis^[134]^. Additionally, related studies have explored different administration routes. For instance, in a study by Zhang *et al.*, the peptide hormone IP-673 promoted the use of recombinant probiotic strain *L. plantarum* strains expressing the HA1 protein, which were administered both orally and intranasally. Mice challenged with a lethal dose of the H1N1 virus showed improved immunization when the administration was intranasal^[135]^. Huynh *et al.* demonstrated HA1 subunit and Bacillus subtilis poly gamma-glutamate synthetase A (pgsA) fusion proteins on the surface of *Lactobacillus casei* L525 and they compared immune response intensities between intranasal and oral administration routes, finding both routes provided similar immune protection, with intranasal administration proving more effective. Several factors, including genetic diversity among *Lactobacilli*^[136]^, antigen characteristics, and mucosal immunization sites, complicate the interpretation of immune outcomes and hinder comparative studies. Table 1 provides a summary of selected probiotics engineered for use as mucosal vaccines [Table 1].

**Table 1:** Some probiotics are designed as mucosal vaccines

**Strains**	**Expression vector**	**Route of vaccination**	**Targeting pathogens**	**Antigen**	**Response method**	**Ref.**
*Lactococcus lactis* ATCC 11454	pMG36e	Oral	*Clostridioides difficile*	Non-toxic C-terminal receptor binding domains of the toxins TcdA and TcdB	The immunized mice all produced high levels of IgG and IgA antibodies	[137]
*Lactococcus lactis* F17847	pTnis	Endonasal	*Streptococcus pneumoniae*	PspA	Associated with a shift to a Th1-mediated immune response characterized by reduced PspA antibody potency	[127]
*Lactococcus lactis* NZ3900	pNZ8110	Oral	*Helicobacter pylori*	Lpp20 antigen	Significantly elevated serum Lpp20-specific IgG antibody levels	[130]
*Lactobacillus plantarum* NC8	pSIP409	Oral	*Trichinella*, *Trichinella spiralis*	Tsgal	Triggered significant intestinal mucosal sIgA responses and specific systemic Th1/Th2 immune responses	[129]
*Lactococcus lactis* NZ9000	pNZ8148-SAM，plSAM-WAE	Oral	*Helicobacter pylori*	Urease, HpaA, HSP60, NAP	Showed increased levels of antibodies against *Helicobacter pylori*, including IgG and sIgA, and significantly reduced *Helicobacter pylori* colonization	[131]
EcN	pNZ8148	Oral, endonasal	Birch and pollen allergens	Bet v 1 Phl p 1, Phl p 5	Intra-application of EcN-Chim significantly reduced lung inflammation, decreased specific IgE levels, and increased specific IgA and IgG2a levels	[138]
EcN	pEHLYA2-SD	Oral, rectal	HIV	gp41 protein	Secretion of C52-HlyA218 peptide inhibits HIV infection of human peripheral blood mononuclear cells *in vitro*	[139]
EcN Δ*dapB*	pKT-5M2e-HlyABD	Endonasal	Influenza A virus	5M2e antigen	EcN-5M2e induces effective humoral, mucosal, and T cell responses	[140]

PsPA: Pneumococcal surface protein A; Tsgal: *Trichinella spiralis* galactose agglutinin; EcN: *Escherichia coli* Nissle 1917; HIV: human immunodeficiency virus.

Viral infections like high-risk HPV-16 are closely associated with tumorigenesis, implying that recombinant *Lactobacillus* vaccines targeting these viruses may exert antitumor effects. This hypothesis was validated in an experimental animal study conducted by Mohseni *et al.*^[141]^. They utilized the vector pNZ8123-HPV16-optiE7 to express the HPV-16 E7 antigen in the *Lactococcus lactis* NZ9000 strain, which was orally administered to female mice. This approach induced high levels of E7-specific antibodies, along with a robust presence of E7-specific CD4^+^ T helper cells and CD8^+^ T cell precursors, thereby demonstrating potent protection against an E7-expressing tumor cell line (TC-1)^[141]^. In a related study, Li *et al.* engineered *Lactococcus lactis* to co-express HPV-16 E7 protein and IL-12, thereby enhancing mucosal immune activation through intranasal administration. IL-12 functions as an immune adjuvant, stimulating a Th1-type immune response and enhancing IFN-γ production, which in turn boosts cytotoxic T-lymphocyte (CTL) activity and enhances tumor cell killing^[142]^.

In addition to probiotic *Lactococcus lactis*, EcN has demonstrated significant potential for mucosal vaccine development. As early as the last century, the non-fimbrial adhesin AIDA-I associated with diffuse adhesion of enteropathogenic *Escherichia coli* (EPEC) was successfully cloned and expressed, contributing to the AIDA autotransporter system^[143]^. Subsequent studies explored integrating this system into EcN’s cell membrane using synthetic biology, incorporating virulence factor antigens to display heterologous polypeptides on the bacterial surface. This approach aims to activate the host mucosal immune system upon mucosal administration, potentially producing immunoprotective effects^[144]^. On this basis, the plasmid pAIDA1-SP, containing the SARS-CoV-2 spike protein (*SP*) gene, was designed and transfected into EcN. This innovation resulted in the development of a mucosal vaccine against COVID-19, which induced time-dependent increases in specific IgG and IgA antibodies in mice following both oral and intranasal administration^[133,145]^. Antigen presentation on the bacterial surface, facilitated by an AIDA autotransporter or an anchoring matrix, is crucial as it enhances stimulation of the local mucosa-associated lymphoid tissue (lamina propria). In comparable oral administration settings, engineered *Lactobacillus casei* utilized PgsA as an anchoring matrix to fuse K99 and K88 fimbrial proteins, achieving higher levels of specific IgA and IgG antibodies and eliciting effective T cell immune responses. This outperformance contrasts with engineered EcN, where the plasmid was directly inserted into the K88 fimbrial adhesin gene^[146,147]^. In both studies, *Lactobacillus casei* demonstrated higher immunogenicity compared to EcN, highlighting that the choice of chassis bacteria can significantly impact the efficacy of mucosal vaccines.

### Advantages and deficiencies of engineered bacteria in disease treatment

Engineered bacteria play a pivotal role as carriers in drug delivery systems, providing a promising platform to enhance the precise delivery of drugs or target molecules to lesions for targeted therapy. This development is particularly significant in treating hypoxic solid tumors such as melanoma, where conventional radiotherapy and chemotherapy often struggle due to their low tumor-targeting efficiency and limited tissue penetration. Engineered attenuated *Salmonella typhimurium*, capitalizing on its facultative anaerobic nature, exhibits a remarkable ability to target and colonize hypoxic tumor tissues. Moreover, releasing mPD-1 modulates immunity, leading to a substantial reduction in tumor volume^[97]^. Additionally, strategies such as surface modification, ecological niche competition, and genetic engineering further augment the efficacy of these targeting approaches^[148]^.

In the treatment of IBD, conventional high-dose administration of anti-inflammatory factors such as IL-10, IL-27, or IL-35 is expensive and often results in significant systemic side effects^[149]^. However, engineered *Lactococcus lactis* can locally produce IL-10 in the intestine upon topical application, effectively reducing systemic side effects associated with high-dose treatments^[35]^. Moreover, probiotics themselves can modulate intestinal microecology and promote mucosal repair, thereby enhancing the effectiveness of engineered bacteria^[150]^. Engineered bacteria also address challenges related to the short half-life of certain drug molecules. For example, in the treatment of neurodegenerative diseases and diabetes mellitus, engineered EcN and *Lactobacillus lactis* can consistently and efficiently express GLP-1, which reduces drug dosage, minimizes side effects, and improves patient adherence^[111,151]^. Furthermore, patients with PKU often face a reduced quality of life due to strict Phe restrictions. Traditional treatments involve daily subcutaneous injections of recombinant PAL, which are effective in some patients but can induce severe allergic reactions^[152]^. Engineered bacterium SYNB1618, expressing PAL, presents a promising solution with demonstrated safety and tolerability in clinical trials^[76]^.

While natural and engineered bacteria hold promise for future disease treatments, studies have highlighted safety concerns associated with these live drugs. Engineered bacterial therapies must address both biosafety and ethical concerns to ensure that live therapeutic bacteria do not spread or undergo unintended recombination in the environment. Antibiotic resistance genes can be carried on mobile genetic elements such as plasmids and transposons, facilitating their exchange through conjugative transfer between bacteria. This horizontal transfer of resistance genes poses a significant risk. For instance, vancomycin resistance genes have been observed to transfer from *Enterococci* to *Lactobacillus acidophilus* even without antibiotic selection pressure, potentially occurring within the human gastrointestinal tract^[153]^. In a study assessing eight commercially available probiotic preparations containing *Lactobacillus acidophilus*, various resistance genes - including those for vancomycin, ciprofloxacin, broad-spectrum β-lactamases, and tetracycline - were detected^[154]^. Without stringent screening, these probiotics could inadvertently serve as vectors for antibiotic resistance genes.

When developing engineered bacteria using plasmid expression systems, the introduction of plasmids carrying exogenous DNA into host bacteria typically involves screening for successful transformation using antibiotic resistance genes. However, the inclusion of these resistance genes in engineered bacteria poses a barrier to their clinical application. To address this issue, researchers are exploring alternative selection markers such as sugar utilization genes, phage resistance factors, and stress tolerance mechanisms. Additionally, emerging gene editing technologies like the CRISPR-Cas system offer a promising avenue for genome editing in bacteria, enabling the creation of antibiotic-free engineered bacteria^[155]^. Currently, most synthetic biology tools are primarily developed for EcN and *Lactobacillus*, with relatively few engineering tools available for strict anaerobic bacteria found in the colon^[156]^. Additionally, the regulatory framework for engineered bacteria as novel therapeutics remains incomplete, and regulatory requirements may vary across countries and regions, presenting a challenge for global clinical trials and product launches.

## SYNTHETIC BIOLOGY AND CRISPR/CAS SYSTEM

The synthesis of proteins and DNA, combined with the establishment of biocentric laws, laid the foundation for the idea of creating living organisms from scratch, leading to the rapid development of synthetic biology. Synthetic biology is an interdisciplinary field that integrates concepts and techniques from biology, engineering, computer science, and molecular biology. It is typically defined as the redesign or engineering of existing biological components and systems using specific technologies to achieve a desired function^[157]^. In 2010, Gibson *et al.* created the first synthetic genomic cell, employing a bottom-up approach in which chemically synthesized DNA fragments were assembled to form a minimal genome necessary for the basic functioning of an organism^[158]^. This minimal genome offers advantages in stability and efficiency, but challenges remain, such as the difficulty of synthesizing long DNA sequences from scratch^[159]^. In contrast, the field of engineered bacteria often adopts a top-down strategy, which involves modifying existing organisms by removing non-essential genes from their genomes. This process frees up space and energy to optimize the expression of target genes^[160]^. For example, studies have shown that heterologous protein production in *Lactobacillus lactis* NZ9000 is significantly improved when 2.83% of the non-essential genome is deleted using the Cre-loxP deletion system^[161]^. Plasmid-based expression systems are commonly used in engineered bacteria, but they face limitations for clinical applications due to issues with genetic instability and the potential for biological contamination. As a result, gene-editing technologies, particularly those based on the CRISPR-Cas system, are emerging as powerful tools. These technologies have the potential to stabilize and modify probiotic genomes, enabling the alteration of their metabolic pathways and biological properties for more precise and reliable applications.

The CRISPR/Cas system is an acquired immune mechanism found in most archaea and approximately half of all bacteria, where it functions to recognize and excise invading viral DNA^[162]^. This system consists of two main components: the sequence responsible for encoding Cas-related proteins and the CRISPR array, which contains a series of repeats interspersed with spacer sequences. The spacer sequences store fragments of foreign genetic material, allowing the organism to “remember” previous invaders^[163]^. The mechanism operates through three main stages: Adaptation: The complex formed by the Cas1 and Cas2 proteins recognizes foreign genetic material and integrates it into the spacer sequences of the CRISPR array. Expression: The CRISPR array is transcribed into pre-crRNA, which is then processed into mature crRNA by Cas proteins. Interference: The crRNA binds to the Cas protein to form a complex that identifies and cleaves foreign DNA sequences that are complementary to the spacer sequence^[164]^. This recognition occurs only when the crRNA binds to the protospacer adjacent motif (PAM) sequence, a short DNA motif located adjacent to the target sequence^[165]^. Based on this mechanism, the CRISPR/Cas system has been developed into a powerful tool for gene editing. Unlike conventional technologies such as zinc finger nucleases (ZFNs) and transcription activator-like effector nucleases (TALENs), which rely on protein-DNA recognition, CRISPR/Cas utilizes base-pairing between complementary RNA and DNA to target specific sequences. This makes it more accurate and efficient^[166]^. Furthermore, CRISPR/Cas technology only requires the design of guide RNAs (gRNAs) to target specific DNA sequences, making it simpler than designing the specific proteins needed for ZFNs and TALENs^[167]^. Once the designed gRNA-Cas complex creates a DNA double-strand break (DSB) at the target site, the host cell can repair the break through either the non-homologous end joining (NHEJ) or homology-directed repair (HDR) pathways^[168]^. Recently, DSB-independent CRISPR/Cas technologies have also been developed, including base editing (BE), prime editing (PE), and CRISPR-associated transposases (CAST), which offer more precise methods for genome modification without causing DSBs^[169-171]^.

Unlike plasmid-based overexpression systems, CRISPR/Cas gene editing allows for precise deletions, additions, and replacements of a strain’s genome, offering a solution to the genetic instability associated with plasmids and the contamination from antibiotic resistance genes. This approach is poised to become a focal point in the development of engineered probiotics. We first examined the application of CRISPR/Cas technology in two major genera of probiotics - *Lactobacillus* and *Bifidobacterium*. Our genomic analysis revealed that CRISPR repeats were present in 59.7% of *Lactobacillus* genomes and 57% of *Bifidobacterium* genomes^[172,173]^. Specific subtypes and sequences were identified, laying the groundwork for future gene editing efforts. For example, Song *et al.* designed a plasmid, pLCNICK, which carries the Cas9D10A enzyme and a sgRNA expression cassette, along with the homology arms of the target gene. This plasmid was introduced into *Lactobacillus casei* via electrotransformation. Upon guiding Cas9D10A to induce a double-stranded break at the target site, homologous recombination repair allowed for the replacement or knockout of the target gene. The efficiency of this gene knockout ranged from 25% to 62%^[174]^. Alternatively, gene knockout can be achieved by obstructing RNA polymerase function with sgRNAs and partially inactivated dCas9 (which cannot cleave DNA), thus creating a physical barrier to transcription^[175]^.

Endogenous CRISPR/Cas systems, in contrast to exogenous ones, offer higher specificity and lower cytotoxicity, which could make them more suitable for use in probiotics^[176]^. Numerous endogenous CRISPR/Cas systems are being developed for application in probiotics. As an example, researchers reprogrammed the endogenous type I-G CRISPR-Cas system in *Bifidobacterium animalis* subsp. *lactis* to identify naturally occurring large deletions and generate a 500-bp deletion in the *tetW* gene, thereby eliminating tetracycline resistance^[173]^. The translation of uridine phosphate ribose transferase in *Bifidobacterium breve* FJSWX38M7 was successfully terminated by a single base substitution and the insertion of three stop codons, using the endogenous Type I-C CRISPR-Cas system of *Bifidobacterium breve*^[177]^. The endogenous Type I-E CRISPR-Cas system was identified in *Lactobacillus crispatus* NCK1350, with Cas3 as its signature protein. After identifying 5’-AAA-3’ as the PAM sequence, the researchers designed the pTRK1183 plasmid containing the specific crRNA. This plasmid successfully facilitated gene deletion (100% efficiency), stop codon insertion (36% efficiency), and single nucleotide substitution (19% efficiency) in *Lactobacillus crispatus* NCK1350^[176]^. Although CRISPR/Cas-based gene editing is still in its developmental phase, significant progress is being made, and several tools are under development. Notably, there are few reports on the direct introduction of large heterologous protein segments into probiotic genomes. A recent study, however, demonstrated successful expression of the HIV-1 membrane-proximal external region (MPER) on the surface of EcN using CRISPR/Cas9. This breakthrough suggests the potential of engineered probiotics to enhance mucosal immune responses^[178]^. As research into CRISPR/Cas continues, it will deepen our understanding of the genetic characteristics of probiotics and open new avenues for their application. Table 2 summarizes the application of CRISPR-Cas-based gene editing technology in some probiotics [Table 2].

**Table 2:** CRISPR-Cas system in some probiotics

**Probiotics**	**Gene editing tools**	**Application**	**Ref.**
*Lactococcus lactis* NZ9000	Cas9 protein, sgRNA, Red/ET recombinase system, pMG36e plasmid	Constructed pMG-Cas9-ldh recombinant plasmid to knock out lactate dehydrogenase gene; developed a food-grade gene editing system to avoid the introduction of antibiotics and exogenous genes	[179]
*Lactobacillus rhamnosus* GG	Cas9, sgRNA, HMME, ZIF-8	Self-driving CRISPR/Cas9 nanosystems can reprogram TIME through multiple pathways	[180]
*Lactobacillus paracasei* NCBIO01-M2	Cas9, sgRNA, pNcas	Obtained a thermotolerant strain NCBIO01-M2-ldhL1-HT capable of efficiently producing L-lactic acid at 45 ℃	[181]
*Lactobacillus reuteri* ATCC PTA 6475	Cas9, tracrRNA, RecT, crRNA, pVPL3017, pVPL3004	CRISPR-Cas9-assisted recombination techniques can facilitate targeted codon saturation mutations. Additionally, CRISPR-Cas9-based selection enables the identification of mutations that occur at low frequencies, such as those resulting from oligonucleotide-mediated chromosomal deletions	[182]
*Bifidobacterium animalis subsp. lactis*	CRISPR-CBE	A large segment deletion was produced in *Bifidobacterium animalis subsp. lactis* using the endogenous CRISPR-Cas system; the CRISPR-cytosine base editor was able to introduce SNPs in *B. lactis*	[173]
EcN	Cas3, crRNA, λ-RED recombination system, cascade protein	Engineered EcN is effective in removing multiple ARGs under *in vitro* and *in vivo* conditions	[183]
EcN	Cas9, sgRNA, pCas9-KT	Removal of two cryptic plasmids, pMUT1 and pMUT2, from EcN reduces metabolic burden; used for GABA production in an antibiotic-free system	[184]
EcN	CRISPR-Cas9 plasmid, pDA, ROS-responsive linker	For targeted delivery of the CRISPR-Cas9 system to deep-seated tumors for photothermal sensitization immunotherapy, liposomes (Lipo-P) loaded with CRISPR-Cas9 plasmid were used to reduce the thermotolerance of tumor cells through gene editing	[185]

HMME: Hematoporphyrin monomethyl ether; TIME: tumor immunosuppressive microenvironment; CRISPR-CBE: CRISPR-Cas9 gene editing system, base editor; SNPs: single-nucleotide polymorphisms; EcN: *Escherichia coli* Nissle 1917; ARGs: antibiotic resistance genes; GABA: γ-aminobutyric acid; pDA: polydopamine; ROS: reactive oxygen species.

Advances in artificial intelligence (AI) are expected to significantly impact research in the field of engineered bacteria, particularly in the areas of editing, screening, and optimizing engineered strains. For instance, the development of the GEDpm-cg platform offers an efficient, user-friendly, and flexible tool for genome editing in *C. glutamicum*, which is anticipated to enable large-scale mutation analysis through robot- and software-assisted systems^[186]^. This platform aims to improve the understanding and engineering of cellular metabolism. Moreover, structural biology plays a key role in the optimization of gene editing tools. In particular, understanding the high-resolution structure of nucleases such as Cas9 is crucial for elucidating how these enzymes recognize and cleave DNA. By analyzing the structure of Cas9, modifications can be made to enhance its specificity and reduce off-target effects.

## CONCLUSION

Natural probiotics have demonstrated beneficial properties such as improving intestinal microbiota, regulating metabolism, and exhibiting antitumor effects. However, nonspecific probiotic supplementation and FMT may lead to variable efficacy and potential safety concerns, making the development of engineered bacteria for more targeted treatments a more sensible approach. Leveraging synthetic biology, researchers are genetically engineering these probiotics to carry out specific functions beneficial to human health. Engineered bacteria show promising potential for treating conditions such as IBD, metabolic disorders, neurodegenerative diseases, and cancer. Despite successful results in animal models, clinical trials are limited, with few products like SYNB1618 demonstrating safety and tolerability^[76]^. None have yet been approved for market use. Advancements in gene editing tools enable the design of complex genetic circuits to finely tune the biological capabilities of engineered bacteria. This includes enhancing their targeting specificity^[187]^ and optimizing the secretion of desired products^[188]^. Despite their therapeutic promise, challenges remain, such as improving safety profiles, refining clinical trial methodologies, and advancing technological innovations. Exploring new applications within the realm of synthetic biology offers avenues for further development and application. In the future, advanced technologies emerging from synthetic biology, structural biology, and AI are expected to significantly contribute to research in the field of engineered bacteria.

## DECLARATIONS

### Copyright

© The Author(s) 2024.
